# Host genetic basis of COVID-19: from methodologies to genes

**DOI:** 10.1038/s41431-022-01121-x

**Published:** 2022-05-27

**Authors:** Kristina Zguro, Chiara Fallerini, Francesca Fava, Simone Furini, Alessandra Renieri

**Affiliations:** 1 Med Biotech Hub and Competence Center, Department of Medical Biotechnologies, University of Siena, Siena, Italy; 2 Medical Genetics, University of Siena, Siena, Italy; 3 Genetica Medica, Azienda Ospedaliero-Universitaria Senese, Siena, Italy

**Keywords:** Genetics, Computational biology and bioinformatics

## Abstract

The COVID-19 pandemic caused by the severe acute respiratory syndrome coronavirus-2 (SARS-CoV-2) is having a massive impact on public health, societies, and economies worldwide. Despite the ongoing vaccination program, treating COVID-19 remains a high priority; thus, a better understanding of the disease is urgently needed. Initially, susceptibility was associated with age, sex, and other prior existing comorbidities. However, as these conditions alone could not explain the highly variable clinical manifestations of SARS-CoV-2 infection, the attention was shifted toward the identification of the genetic basis of COVID-19. Thanks to international collaborations like The COVID-19 Host Genetics Initiative, it became possible the elucidation of numerous genetic markers that are not only likely to help in explaining the varied clinical outcomes of COVID-19 patients but can also guide the development of novel diagnostics and therapeutics. Within this framework, this review delineates GWAS and Burden test as traditional methodologies employed so far for the discovery of the human genetic basis of COVID-19, with particular attention to recently emerged predictive models such as the post-Mendelian model. A summary table with the main genome-wide significant genomic loci is provided. Besides, various common and rare variants identified in genes like *TLR7, CFTR, ACE2, TMPRSS2, TLR3*, and *SELP* are further described in detail to illustrate their association with disease severity.

## Introduction

Severe acute respiratory syndrome coronavirus-2 (SARS-CoV-2) and consequent COVID-19 have resulted in a serious threat to human health and public safety. For almost 2 years now, both scientists and clinicians have been trying to understand why a large group of individuals are asymptomatic, while others undergo life-threatening viral pneumonia and acute respiratory distress syndrome. Age, sex, and comorbidities are relevant clinical variables in determining the response to SARS-CoV-2 infection [1]. Nevertheless, these risk factors do not explain the severity of COVID-19, particularly in healthy young subjects.

**Fig. 1:**
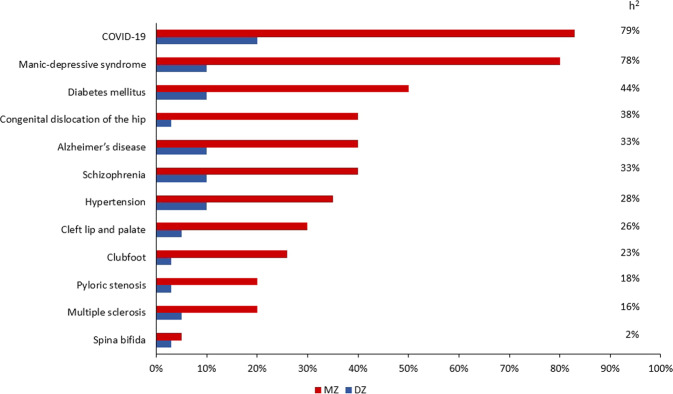
Twin concordance. Estimated monozygotic (MZ) and dizygotic (DZ) twin concordance rates for various medical disorders. The percentage referred to the inheritance (*h*^2^) for each condition has been calculated using the formula: *h*^2^ = (*C*_MZ_ – *C*_DZ_) / (1 – *C*_DZ_). In each multifactorial trait, the concordance rate in MZ twins exceeds that in DZ twins. The demonstrated percentages reflect the heritability of the conditions: the higher the monozygotic concordance, the more important the genetic contribution, and the higher the heritability. COVID-19 seems to have a high heritability with a concordance rate of 80% in MZ twins. Nevertheless, these observations were derived from a recent study performed on 10 pairs of young twins [3]. Thus, further studies in larger sample sizes are needed to better evaluate the precise heritability of COVID-19. Modified from [49]. The heritability (in percentage) of COVID-19 reported here was calculated considering the twin pairs of the study [3] and another MZ twin pair mentioned in the same paper [3].

COVID-19 has demonstrated itself to be a complex multifactorial disease, but its main environmental factor (SARS-CoV-2) is easily detectable by a PCR-base swab test. Thus, it represents an accessible disorder for identifying the role of human genetics in susceptibility to infection. Indeed, classical twin studies have already stressed the fact that there is a genetic component associated with the highly varied clinical outcomes of COVID-19. A research team, based on data from over 3000 TwinsUK volunteers completing the C-19 symptoms tracker app, found a substantial genetic influence for delirium (heritability of 49%), diarrhea (heritability of 31%), fatigue (heritability of 31%), anosmia (heritability of 19%), and for predicted COVID-19 (heritability of 31%) [2]. Moreover, a recent work compared the concordance rate in 10 pairs of young twins, 5 monozygotic (MZ), and 5 dizygotic (DZ), and reported a higher concordance rate in the MZ group (83%), further supporting the significant role of the genetic make-up in the variable clinical manifestations of COVID-19 [3] (Fig. 1). On these bases, several methods have been employed to reveal the genomic determinants of COVID-19 susceptibility and severity. The classical approach, based on genome-wide association studies (GWAS), has identified some common polymorphisms in relevant genes [4–8], while the Burden test, focused only on rare coding variants, has not identified any significant associations until recently [8, 9]. On the other hand, it is worth emphasizing the fast-growing role of machine learning (ML) models in classification or clustering tasks in genomic datasets [10]. One can expect that the latter will help in resolving the genetic variation underlying COVID-19 by combining rare and common variants into an overall predictive model.

Despite the ongoing vaccination programs and other preventive measures, treating the disease remains a high priority. Thus, delineating the virus-host interactions will be crucial to elucidate further COVID-19 pathogenesis and to translate these findings to improve patient care and further drug developments for new virus variants as they arise.

This review summarizes the main approaches used thus far for unbiased gene discovery and highlights relevant identified genes associated with susceptibility or severity to COVID-19. While the data reported here are definitely relevant for discussing COVID-19 prevention and treatment, this review will focus mainly on methodology and disease mechanism, leaving therapeutic aspects to another specific review.

## How to study genetic susceptibility of COVID-19—methodologies for unbiased gene discovery and model predictivity

### GWAS

The most robust and traditional approach for gene discovery is GWAS [11]. GWAS study single-nucleotide polymorphisms (SNPs) that are reported as clusters of correlated variants demonstrating a statistically significant association with complex disorders.

These studies require sample sizes of ten/hundred thousand subjects to have sufficient statistical power to detect a moderate association while analyzing hundreds of thousands to millions of SNPs Predominately, GWAS focus mainly on common variants, usually with a minor allele (MAF) ≥5% whose effects are relatively small. However, the inclusion of variants with a frequency of up to 1% can be achieved.

The GWAS approach is based on a straightforward comparison of about 700,000 genomic SNPs frequencies in cases/controls. Over 90% of GWAS variants fall in non-coding regions of the genome and therefore do not directly affect the coding sequence of a gene. Thus, deeper follow-up analyses are needed to pinpoint the relevant genes. The coverage of the coding SNPs is usually performed throughout imputed data, e.g., imputing 2 million SNPs from 700k SNPs by linkage disequilibrium. A major limitation of genome-wide approaches is the necessity to choose a high level of significance, *p* < 5 × 10^−8^, because of the multiple independent tests. Ultimately, the “missing heritability” problem of GWAS can be partially explained by rare variants.

**Fig. 2:**
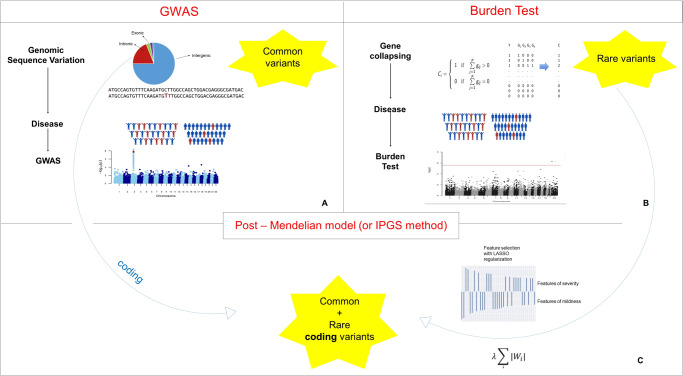
Methodologies for features selection. **A** Genome-wide association studies methodology for the study of SNPs. **B** Burden test methodology for the study of rare coding variants. **C** Post-Mendelian model for the study of both common and rare coding variants.

GWAS for COVID-19 have been facilitated by international collaborations [12] that are sharing scientific methods and resources to shed light on the genetic determinants of SARS-CoV-2 infection and the outcomes of the resulting disease. Up to date, multiple GWAS have successfully identified various genome-wide significant loci associated with some aspect of SARS-CoV-2 or COVID-19 (Fig. 2A) [4–8].

### Burden test

Since GWASs focus on identifying common variants, it is probable that the analysis of rare variants (MAF < 0.5%) could further contribute to clarifying the role of rare genetic determinants in the etiology of SARS-CoV-2 infection. In this context, another robust and traditional method is the Burden test [13]. This approach is based on an aggregation of rare, protein-altering variants and a comparison between case and control subjects. The reasoning behind the burden testing is that grouping variants with a large effect size at a gene level might improve power. Like GWAS, the Burden test method needs hundreds of thousands of participants to detect statistically significant associations. Thus far, many working groups have tried to characterize rare variants and explain the biological mechanism of patients with severe COVID-19, but with no considerable proof of association yet (Fig. 2B) [8, 9].

## How many genes are expected to be involved in complex disorders?

Both GWAS and Burden tests were able to identify some tens of genes that were not sufficient to explain the heritability of the disease and to fully predict severity. New methodologies able to identify the entire genetic variability, and combine both common and rare variants are necessary.

When talking about complex diseases, height is the archetype of polygenicity [14]. Such complex traits are products of many genes which interact together in a complex way. Hundreds of common variants, as well as rare and low-frequency variants, have been reported to be associated with height [14, 15]. As stated by Boyle et al. [14], the disease risk is mostly determined by genes not directly relevant to the disease and by a much smaller number with direct effects. The authors suggested that genetic features for complex traits could reach thousands or even hundreds of thousands.

COVID-19 is a complex multisystem disorder, and as such, a much greater number of genes are expected to be involved; much greater than the tens reported by GWAS and Burden tests.

### Post-Mendelian model

Methods neglecting the combined contribution of common and rare variants were unlikely thus far to thoroughly characterize the host genetics underlying COVID-19. Thus, new ML methods are under development [16–18].

**Fig. 3:**
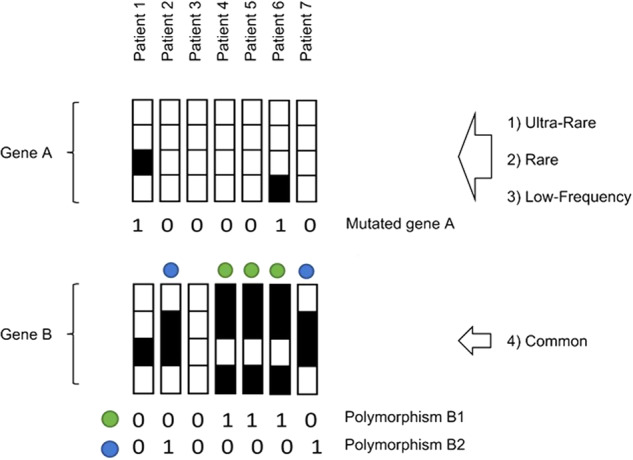
Boolean representation of genetic variants in the post-Mendelian model. The upper chart demonstrates that the feature “Mutated gene A” is defined by considering possible variants (ultra-rare, rare, and low-frequency) of gene A. The feature “Mutated gene B” is defined by the combination of two or more different common coding variants.

One of these novel predictive models is the Post-Mendelian model which was proposed to aggregate the effects of all genetic components into a score, named Integrated PolyGenic Score (IPGS) [16, 17]. The main steps necessary for the definition of this IPGS were: (i) the representation of the genetic variability into a separate set of Boolean features, representing variants of different frequencies and different models of heritability; (ii) the selection of the features more likely to be predictive for the clinical phenotype; and (iii) finally the identification of the weighting factors required to combine common and rare variants into a unique score. As described in Fig. 3, the variants were binarized into 0 or 1 based on the absence or presence of variants in each gene. In the case of common polymorphisms, 1 corresponds to different combinations. The representation of the genetic variability by Boolean features responds to two requirements. Firstly, the usage of summary features at the gene level widely reduces the number of input features. Moreover, this combination of single genetic variants into gene-level variables highly facilitates the interpretation of the results. Interpretability is an important characteristic of ML models for predicting COVID-19 phenotypes, as only an easily interpretable model can be useful in clinical practice and significantly contribute to diagnostic and therapeutic targeting. The total number of input features with binary classification is much lower than the number of genetic variants, but still, Boolean features vastly outnumber the number of individual patients. Logistic regression models with L1 regularization were used to identify the most important subset of input features for predicting the clinical phenotype. In L1 regularization, model parameters are forced to be zero for any parameter associated with an input feature that is not significantly predictive of the target variable (as the derivative of the cost function with respect to a model parameter does not depend on the magnitude of the parameter). This procedure identified around 8000 features, corresponding to around 4000 involved genes. This high number of genes is in sharp contrast with the results described in previous sections regarding GWASs and Burden test. However, it should be noted that the aims of these three methods are also different. The scope of the GWASs and Burden test is to identify variants that are associated with the phenotype with some statistical significance. Instead, the gene selection procedure described here identifies a large set of genes likely to be predictive of the clinical phenotype. The statistical significance is tested for the clinical predictions of the model (i.e., testing that the final model is statistically more predictive than a model not including IPGS), not for each single input feature selected. For the definition of a predictive score combining common and rare variants, it is important to keep in mind the observation that variants at different frequencies are expected to contribute differently to the phenotype, almost by definition. The weighting factors of the various frequency terms were estimated by optimizing the separation between mild and severe cases provided by the IPGS.

To sum up, the final testing of the model in independent cohorts proved that a model including IPGS was statistically more predictive than a model predicting the severity from age and sex alone. Furthermore, the high number of predicted genes (**≈**4000) might be consistent with the “omnigenic” model of complex traits introduced by Boyle et al. In this context, the results of the post-Mendelian model might offer good bases for further investigation on peripheral genes, as well as explain the missing heritability in COVID-19 (Fig. 2C).

### Genes involved in COVID-19

As in all infectious diseases, besides the important role of pathogen genetics, host genetics and physiology are essential elements in defining the clinical course of disease in COVID-19 patients. Numerous studies have identified thus far several human genetic variants that contribute to different responses to SARS-CoV-2 infection.

### GWASs and eQTL

Up to date, multiple GWASs have successfully identified various genome-wide significant loci associated with clinical phenotypes of COVID-19 susceptibility/severity that are summarized in Table 1.

**Table 1:** GWAS loci in COVID-19.

Locus	chr:pos (hg38)	REF	ALT	rs	OR	Consequence	Genes	References
1	1:155066988	C	T*	rs114301457	2.40	Synonymous	*EFNA4*	[7, 8]
2	1:155175305	G	A*	rs7528026	1.39	Intron	*TRIM46*	[7, 8]
3	1:155197995	A*	G	rs41264915	1.20	Intron	*THBS3*	[7, 8]
4	2:60480453	A*	G	rs1123573	1.13	Intron	*BCL11A*	[7, 8]
5	3:101705614	T*	C	rs11919389	1.02	Downstream	*ZBTB11, RPL24, CEP97, NXPE3*	[6]
6	3:146517122	G	A*	rs343320	1.25	Missense	*PLSCR1*	[7, 8]
7	3:45796521	G	T*	rs2271616	1.26	5’UTR	*SLC6A20*	[7, 8]
	3:45823240	T	C*	rs10490770	1.88	Downstream	*LZTLF1*	[6]
	3:45834968	AA	AAA*	rs11385942	1.77	Intron	*SLC6A20, LZTFL1, FYCO1, CXCR6, XCR1, and CCR9*.	[4]
	3:45859597	C	T*	rs73064425	2.71	Intron	*LZTLF1*	[7, 8]
8	5:131995059	C	T*	rs56162149	1.20	Intron	*ACSL6*	[7, 8]
9	5:132441275	T	C*	rs10066378	1.20	Intron	*IRF1-AS1*	[7]
10	6:41534945	A	C*	rs1886814	6.17	eQTL, Intron	*FOXP4*	[6, 22]
11	6:29831017	A*	G	rs9380142	1.3	3’UTR	*HLA-G*	[5]
12	6: 32180146	A	G*	rs3131294	1.1	Intron	*NOTCH4*	[5]
13	6:32623820	T*	C	rs9271609	1.14	Upstream	*HLA-DQA1*	[7]
14	6:41515007	A*	C	rs2496644	1.45	Intron	*LINC01276*	[7]
15	8:124324323	T	C*	rs72711165	1.227	Intron	*TMEM65*	[6]
16	9:133257521	A	C*	rs657152	1.32	Intron	*ABO*	[4]
	9:133274084	C*	T	rs912805253	1.14	eQTL, Intron	*ABO*	[6, 22]
17	11:34482745	G*	A	rs61882275	1.27	Intron	*ELF5*	[7, 8]
18	9:21206606	C	G*	rs28368148	1.45	Missense	*IFNA10*	[7]
19	12:113363550	C	T*	rs6489867	1.3	Intron	*OAS1–OAS3*	[5]
	12:112919388	G	A*	rs10774671	1.2	eQTL, Splice Acceptor Variant	*OAS1–OAS3*	[6, 22]
	12:113380008	G	A*	rs10735079	1.3	Intron	*OAS1–OAS3*	[5]
20	12:132489230	GC	G*	rs56106917	1.13	eQTL, Upstream	*FBRSL1*	[7, 8]
	12:132479205	G	A*	rs4883585	1.13	Upstream	*FBRSL1*	[7]
21	13:112889041	C	T*	rs9577175	1.18	Downstream	*ATP11A*	[7, 8]
22	15:93046840	T*	A	rs4424872	1.04	Intron	*RGMA*	[7, 8]
23	16:89196249	G*	A	rs117169628	1.18	eQTL, Missense	*SLC22A31*	[7]
24	17:46142465	T*	A	rs1819040	1.1	eQTL, Intron	*ARHGAP27, PLEKHM1, LINC02210-CRHR1, CRHR1, SPPL2C, MAPT, STH, KANSL1, LRRC37A, ARL17B, LRRC37A2, ARL17A, NSF, WNT3*	[6, 22]
25	17:49863260	C	A*	rs3848456	1.50	regulatory	*–*	[7, 8]
26	17:49863303	C	T*	rs77534576	1.45	Downstream	*KAT7, TAC4*	[6]
27	19:10466123	T	C*	rs11085727	1.3	eQTL, Intron	*TYK2*	[5, 22]
	19:10317045	T	A*	rs74956615	1.6	eQTL, Downstream	*ICAM1, ICAM4, ICAM5, ZGLP1, FDX2, RAVER1, ICAM3, TYK2*	[5, 6]
	19:10352442	G*	C	rs34536443	1.5	eQTL, Missense	*TYK2*	[7, 8]
	19:10305768	G	A*	rs73510898	1.24	eQTL, Intron	*ZGLP1*	[7, 8]
28	19:4719431	A	G*	rs2109069	1.4	Intron	*DPP9*	[5]
	19:4717660	A	G*	rs12610495	1.3	Intron	*DPP9*	[7, 8]
29	19:48697960	C	T*	rs368565	1.15	eQTL, Intron	*FUT2*	[7, 8]
30	19:48867352	G*	T	rs4801778	1.1	Intron	*PLEKHA4, PPP1R15A, TULP2, NUCB1*	[6]
31	21:33230000	C	A*	rs17860115	1.24	eQTL, 5’UTR	*IFNAR2*	[7, 8, 22]
	21:33242905	T*	C	rs13050728	1.23	eQTL, Intron	*IFNAR2*	[6, 22]
32	21:33287378	C	T*	rs8178521	1.18	eQTL, Intron	*IL10RB*	[7, 8, 22]
33	21:33959662	T	TAC*	rs35370143	1.26	Intron	*LINC00649*	[7, 8]
	21:33914436	A	G*	rs12626438	1.22	Intron	*LINC00649*	[7]

*REF* reference allele, *ALT* alternate allele, *OR* odds ratio of the risk allele.

Asterisk (*) corresponds to the risk allele.

The first GWAS detected the 3p21.21 locus (rs11385942) and 9q34.2 locus (rs657152) with a significance at the genome-wide level of *p* < 5 × 10^−8^ [4]. The 3p21.21 locus was associated with severe COVID-19 and respiratory failure, while the association signal at locus 9q34.2 coincided with the *ABO* blood group locus. In the cohort of this study, a blood-group-specific analysis was further performed, which showed a higher risk in the A blood group, and a protective effect in the O blood group compared with other blood groups.

Another paper within the GenOMICC study was performed on critically ill patients with COVID-19. The association signals discovered were at locus 12q24.13 (rs10735079), 19p13.2 (rs74956615); 19p13.3 (rs2109069); and 21q22.1 (rs2236757) all with a significance of *p* < 5 × 10^−8^. These signals were additionally replicated and linked with life-threatening COVID-19 [5].

A more recent study brought together the largest number of COVID-19 host genetics studies thus far employing standardized methods [6]. This case–control GWAS meta-analysis of 46 studies from 19 countries identified 13 distinct loci associated with SARS-CoV-2 infection or COVID-19 with a significance of *p* < 1.67 × 10^−8^. The strongest signal for increased susceptibility to SARS-CoV-2 infection was at the *ABO* locus, with variants in two additional loci (*PPP1R15A* and *SLC6A20*). Nine loci were associated with an increased risk of developing severe COVID-19 symptoms, including variants in *DPP9* (OR 1.29, *p* = 2.0 × 10^−12^) and *FOXP4* (OR 1.2, *p* = 6.0 × 10^−13^) that were previously shown to increase the risk for interstitial lung disease. The lead variant in *TYK2* (rs74956615) (19p13.2), previously identified as an autoimmune disease-protective variant, conferred an increased risk for hospitalization due to COVID-19 (OR 1.43, *p* = 9.71 × 10^−12^). On the other hand, the intronic variants 1q22 and rs1819040 in *KANSL1* (17q21.31) (OR 0.96, *p* = 1 × 10^−20^) were associated protectively against COVID-19-related hospitalization. Interestingly, the heritability of SARS-CoV-2 infection was enriched in genes expressed in the lung (*p* = 5 × 10^−4^). Overall, this meta-analysis suggests a polygenic architecture of SARS-CoV-2 infection and COVID-19 severity.

The GenOMICC study on *medRxiv* and their latest published work reported 22 replicated genetic associations with severe COVID-19 and 3 additional loci discovered in 7491 critically ill patients [7, 8]. Several variants associated with the life-threatening disease were related to interferon (IFN) signaling, e.g., variants in *IL10RB* (rs8178521) or *PLSCR1* (rs343320). In addition, significant associations were found in several genes implicated in B-cell lymphopoiesis and differentiation of myeloid cells with the strongest fine-mapping signal at 5q31.1 (chr5:131995059:C:T, rs56162149). A new genetic association at 13q14 (rs1278769), in *ATP11A*, has been already reported to be involved in lung disease. Through transcriptome-wide association and colocalization, the researchers found evidence that the reduced expression of the membrane flippase *ATP11A* and increased mucin expression *MUC1* (as the mediator of the association with rs41264915) contribute to the development of critical disease. Ultimately, the set for the *FUT2* locus including the stop-gain, non-secretor allele (rs492602), was shown to be protective against life-threatening COVID-19.

The GWAS conducted up to now have identified variants mainly in the non-coding region of the genome, and therefore potentially involved in gene regulation. The analyses of such variants in a gene expression level have been done through studies on expression quantitative trait loci (eQTLs) in an effort to pinpoint the likely causative gene(s) at the associated loci and consequently to discover the molecular pathways driving disease pathogenesis.

Thus far, eQTL analyses have identified several likely causal variants for the increased/decreased expression of relevant genes associated with COVID-19 severity. For example, rs505922, a trans-eQTL of *CD209* was found to be associated with increased CD209 levels and COVID-19 severity. On the other hand, rs505922 was interpreted as a cis-eQTL of the ABO protein and thus, it was hypothesized that the decreased ABO plasma protein levels might exert protective effects [19]. Another study identified five common variants (rs3787946, rs9983330, rs12329760, rs2298661, and rs9985159) at *TMPRSS2*/*MX1(21q22.3)* locus which were associated with less severe disease [20]. While the key role of *TMPRSS2* in viral fusion is already explained in the above sections, *MX1* is a guanosine triphosphate-metabolizing protein involved in the cellular antiviral response and induced by both type I and III IFN pathways [21]. Of note, all five SNPs showed eQTL signals for *MX1* in blood tissue. Specifically, the minor alleles of the five polymorphisms correlated with an increased level of *MX1* expression and were associated with a reduced risk of developing COVID-19. These results demonstrate that *MX1* might be related to the diverse clinical outcomes of COVID-19 and suggest that its encoded protein could be a potential therapeutic target. Regarding the *OAS* genes, which play an important role in the innate immune response to viral infections, the intronic variant rs4767027 resulted in increasing the expression of *OAS1* and thus decreasing the hospitalization risk [19]. Moreover, a recent study characterized the association between COVID-19 GWAS loci and eQTLs in 69 human tissues identifying colocalization of GWAS and eQTL signals with an expression of 20 genes in 62 tissues [22]. Among them, the rs1886814 of the *FOXP4* gene associated with the severity of COVID-19, colocalized with a lung-specific eQTL leading to an increased *FOXP4* expression.

### Common coding variants

For cell entry, the S protein of SARS-CoV-2 undergoes a two-step cleavage before fusion. The first cleavage occurs between the S1 and S2 domain and it is performed by host cell proteases Tmprss2 and furin. The second cleavage occurs in the S2 domain to allow membrane fusion. The *TMPRSS2* gene variants have been shown to play an important role in the interindividual differences in COVID-19 susceptibility and severity. For example, the variant p.(Val197Met) (rs12329760) emerged as a common variant, with a minor allele frequency of 0.23 (European non-Finnish), in an Italian cohort of 1177 COVID-19 affected patients and it was shown to have a protective effect particularly in young males and elderly women [23]. This missense mutation located at the exonic splicing enhancer has a deleterious effect that weakens Tmprss2 stability. As Tmprss2 protein promotes cellular entry of SARS-CoV-2, a faulty expression of the protease may contribute to asymptomatic or mild patients.

Coagulation abnormalities, like significantly increased levels of P-selectin and other prothrombotic biomarkers, have been already reported in severe COVID-19 patients [24]. P-selectin is a cell adhesion molecule responsible for mediating the interaction of activated platelets with leukocytes. Its involvement in thrombotic events in various conditions has already been described [25]. A recent study performed within the Italian GEN-COVID cohort identified an association between the homozygous state of the functional polymorphism p.(Asp603Asn) (rs6127) in P-selectin gene (*SELP*) and COVID-19 severity in a subcohort of 513 male subjects [26]. Indeed, the *SELP* rs6127 has been already linked with thrombotic risk since, jointly with other coding polymorphisms, it makes the P-selectin more efficient at recruiting leukocytes to the endothelium [27].

Another characteristic observed in severe patients is impaired consciousness, including delirium [28]. On this basis, *ApoE* e4 alleles have been tested in an attempt to find a correlation with COVID-19 severity, as *ApoE* e4 genotype has been associated with both dementia and delirium [29]. Interestingly, individuals homozygous for *ApoE* e4 (rs429358) have twice the risk of severe COVID-19 compared to the most common *ApoE* e3e3 genotype. This increased incidence of severe COVID-19 might be due to the regulation of proinflammatory pathways and lipoprotein function being affected by the *ApoE* e4 genotype [30].

Common coding polymorphisms linked with COVID-19 severity have been identified also in genes of the innate immune system like the *TLR3* gene. Given the protective role of *TLR3* in other infectious diseases [31], an association between the functional variation in its gene and COVID-19 incidence was hypothesized. Specifically, the common missense variant in exon 4, p.(Leu412Phe) (rs3775291) was considered since it has been already reported to affect TLR3 expression and the subsequent activities needed for proper signaling [32]. This variant showed a poor recognition of SARS-CoV-2 dsRNA compared to the wild type in molecular docking analysis, suggesting impaired immune protection. A population-scale study performed a Pearson correlation coefficient analysis on data from 40 countries (*p* value <0.05 was considered significant) to identify a probable genetic association of Toll-like receptor (TLR) mutant rs3775291 with COVID-19 susceptibility, mortality, and percentage recovery [33]. Indeed, this statistical analysis demonstrated that even though there was no correlation between rs3775291 mutant and percentage recovery of COVID-19 patients, there was a significant positive correlation of TLR3 mutant (rs3775291) with SARS-Cov2 susceptibility and mortality due to COVID-19 with *p* values of 0.0137 and 0.0199, respectively. Further evidence for the TLR3 polymorphism rs3775291 was given by a nested case–control study within the Italian GEN-COVID cohort [34]. In this study, the Italian group not only found a prevalence of the variant in cases rather than in controls, but the performed experiments also suggested the importance of autophagy downstream of the TLR3 receptor. An abolished production of TNF-α is translated in absence of autophagy and thus in susceptibility to infections, including SARS-CoV-2.

**Fig. 4:**
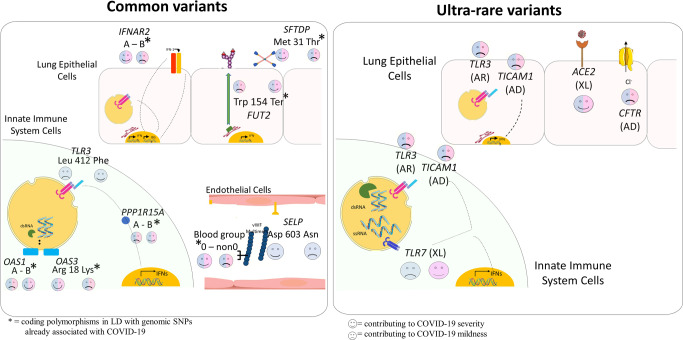
Examples of genes involved in COVID-19 through either common or rare variants. The figure illustrates examples of common (left) and rare (right) variants contributing to either COVID-19 severity or mildness.  = contributing to COVID-19 severity;  = contributing to COVID-19 mildness. Pink faces = contributing to females only; Blue faces = contributing to males only; Pink/Blue faces = contribution in both sexes. In parentheses: AD = autosomal-dominant inheritance; AR = autosomal-recessive inheritance; XL = X-linked recessive inheritance. **A** The common coding polymorphisms p.(Leu412Phe) in the *TLR3* gene and p.(Asp603Asn) in the *SELP* gene were associated with COVID-19 severity. The coding polymorphisms denoted with an asterisk, are in LD with genomic SNPs already associated with critical illness: *SFTDP* gene encoding for SP-D protein, *PPP1R15A* gene encoding for GADD34 protein. *OAS1* haplotype A = c.1039-1G>A, p.(Gly162Ser), p.(Ala352Thr), p.(Arg361Thr), p.(Gly397Arg), p.(Thr358Profs*26). *OAS1* haplotype B = haplotype without the variant combination in haplotype A. **B** Rare mutations in the Toll-like receptors *TLR7*, *TLR3*, and *TICAM1* (encoding TRIF protein), already reported associated with XL, AR, and AD inheritance, impair type I IFN cell-intrinsic immunity. The specific location of *TLR7/8* (on the X chromosome) is responsible for opposite effects in males and females. In lung epithelial cells, *ACE2* rare variants exert protective effects presumably due to lowering virus entrance. Rare variants of the CF-causing rare variants are associated with severity in both sexes.

Some of the above-described genes with common coding variants implicated in COVID-19 are illustrated in Fig. 4 (left panel).

### Rare coding variants

Delineating the role of rare variants in COVID-19 is important to elucidate the pathogenic mechanisms in various subsets of SARS-CoV-2-positive individuals. Thus far, different studies have reported several rare variants that might influence COVID-19 outcomes.

*ACE2* has been a target gene for research works, as it is indispensable for SARS-CoV-2 to enter cells. An early study performed on the Italian population mined whole-exome sequencing data of 6930 Italian controls individuals from five different centers looking for *ACE2* variants [35]. Besides identifying more common variants potentially affecting protein structure, the research group also revealed rare variants that might explain a diverse affinity for the SARS-CoV-2 S protein. Three missense variants p.(Val506Ala), p.(Val209Gly), and p.(Gly377Glu) were predicted to destabilize the protein structure. Likewise, the rare variants namely p.(Pro389His) and the p.(Leu351Val) were predicted to cause conformational changes in ACE2, thus affecting the internalization process of the virus.

Furin protease, ubiquitously expressed, is considered a key player that mediates the maturation of S protein processing and recognition of membrane proteins. This evidence makes furin a crucial molecule for SARS-CoV-2 and ACE2 receptor interaction. In particular, recent data correlates the role of furin protein with severe cardiovascular events in COVID-19 patients, a hypothesis supported by a high level of furin in the peripheral blood of heart failure patients [36]. A variant, p.(Arg298Gln) (rs769208985) was identified in COVID-19 patients among other rare variants in the furin gene *PCSK3* [37]. The amino acid change from arginine to glycine occurred in a very highly conserved position near the substrate-binding residues. In silico analyses showed that the variant might not alter the structure of the protein, but it could affect furin recognition of the SARS-CoV-2 S protein.

**Fig. 5:**
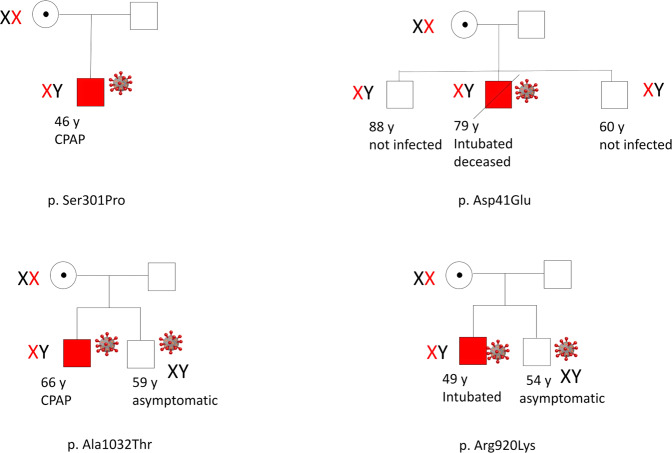
Families affected by severe COVID-19 due to *TLR7*. The disease segregates as an X-linked recessive trait conditioned by the viral infection. Relatives mutated but not yet infected are at risk of severe COVID-19 if infected. Families on the left are reported in Fallerini et al. [40]. Families on the right are reported in Mantovani et al. [50]. The specific *TLR7* mutation is reported at the bottom of each pedigree. red X: chromosome bearing the mutation; symbol of the virus: infected subject. Red symbol = severely affected COVID-19 patients. White symbol = healthy subjects.

If the virus makes it through the target cell, the host immune system recognizes it, eliciting the innate or adaptive immune response. TLRs are key elements in the activation of innate immune responses to a variety of pathogens, generating the production of proinflammatory cytokines such as TNF-α, IL-1, IL-6, and type I and II IFNs. Among different types of TLRs, TLR7 recognizes single-stranded RNA of many viruses including SARS-CoV-2 [38]. In July 2020, van der Made et al. [39] reported rare, deleterious germline variants in the *TLR7*. In this case series of four young men from two unrelated families with severe COVID-19, the identified variants were a maternally inherited 4-nucleotide deletion (c.2129_2132del; p.(Gln710Argfs*18)), and a missense variant (c.2383G>T; p.(Val795Phe)). These unique loss-of-function variants were linked with abrogated production of type I and II IFN responses in the patients’ peripheral blood mononuclear cells when stimulated with the TLR7 agonist, imiquimod. Furthermore, a more recent nested case–control study identified in the *TLR7* gene, loss-of-function variants such as p.(Ser301Pro), p.(Arg920Lys) (rs189681811), and p.(Ala1032Thr) (rs147244662) found in 2.1% of young males with severe COVID-19 [40]. Examples of families affected by severe CVOID-19 due to *TLR7* are depicted in Fig. 5. The corresponding functional gene expression analysis was in line with the previously described study; reduced expression of TLR7 in cases compared to controls and impairment in type I and II IFN responses. These findings elucidate the crucial role of *TLR7* in the recognition of SARS-CoV-2 and in the following elicitation of an early antiviral immune response that could prevent the progress into a severe form of COVID-19.

Besides *TLR7*, inborn errors of type I IFN immunity were found as well to be implicated in the development of a severe form of COVID-19 [41]. The COVID Human Genetic Effort Consortium [42] examined the genetic basis in cases with critical COVID-19 pneumonia and discovered rarely predicted loss-of-function variants in human genes known to regulate TLR3 and the interferon regulatory factor 7 (IRF7)-dependent type I IFN immunity. Specifically, the disease-causing variants were found in the following genes: *TLR3*, *UNC93B1, TICAM1, TBK1, IRF3, IRF7, IFNAR1, IFNAR2*. From 659 tested unrelated patients, at least 3.5% (23) of them suffered autosomal-recessive or autosomal-dominant deficiencies at one of the eight mentioned loci. The results of this study reinforce the key role of TLR3 as a double-stranded RNA sensor and type I IFN cell-intrinsic antiviral immunity in hindering SARS-CoV-2 infection.

Another interesting gene that has been under investigation is *CFTR*. Given that either COVID-19 or cystic fibrosis (CF) affects the respiratory tract, exploring the interaction between both diseases may guide the development of future treatments. A research work performed on a cohort study of 874 Italian individuals diagnosed with COVID-19 identified validated CF-causing variants [43]. The CF carriers represented 8.7% of mechanically ventilated patients and were significantly younger compared to noncarriers with a mean age of 51 and 61.42 years, respectively. These data suggest that the individuals harboring CF-causing variants are more susceptible to the severe form of COVID-19. The latter has also been hypothesized by others [44].

The genes described in this section are depicted in Fig. 4 (right panel).

### Future perspectives

The methods for gene discovery described in this review are based on the simplified assumption that cases and controls are homogeneous. However, several pieces of evidence are suggesting that COVID-19 is not the case. COVID-19 is a systemic disorder involving several organs and tissues, not only the lungs. Hierarchical clustering analysis indicates the presence of different phenotypic clusters among severe cases [45]:

(A) severe multisystemic disease, with either thromboembolic (A1) or pancreatic variant (A2);
(B) cytokine storm, either moderate (B1) or severe with liver involvement (B2);
(C) moderate disease, either without (C1) or with (C2) liver damage;
(D) heart-type, either with (D1) or without (D2) liver damage
(E) Also, mild cases can be divided at least in:
(F) mild disease, either with (E1) or without hyposmia (E2)

Furthermore, it is likely that by shifting the phenotypic level of analysis from the clinical level to laboratory analysis, additional heterogeneity will emerge. For example, concerning the immune system, the group of Prof. Katsikis [46], using a relatively low number of cases, and 17 laboratory variables, was able to identify two different immunophenotypes in severe cases, which are distinct from the immunophenotype of mild cases.

ML methods, considering this heterogeneity, are necessary for further improving the post-Mendelian model. As an example, a possible method that could be used to accomplish this aim is topological data analysis (TDA). TDA is an emerging approach for analyzing high-dimensional data using tools from the mathematical field of algebraic topology [47], which is useful for gaining insights into large-scale datasets, thanks to dimensionality reduction and robustness to noise. By taking into account both geometric and topological characteristics of multi-dimensional data, TDA leads to better results than using traditional analytical methods by preserving the complex relationships within the data and examining them together. This approach has been already used for biological issues but only at the transcriptional level [48], while its use at the genomic level is still unexplored but very promising. With the increase in data availability and a better knowledge of the mechanisms involved in COVID-19 severity, novel approaches for linking severity and susceptibility to the disease to host genetics are likely to emerge.
